# An Efficient One-Pot Synthesis of Pyrano[3,2-*c*]quinolin-2,5-dione Derivatives Catalyzed by L-Proline

**DOI:** 10.3390/molecules171213856

**Published:** 2012-11-22

**Authors:** Songlei Zhu, Jing Wang, Zhou Xu, Jie Li

**Affiliations:** 1 Key Laboratory of Biological Cancer Therapy of Jiangsu Province, Xuzhou Medical College, Xuzhou 221004, China; 2 Department of Chemistry, Xuzhou Medical College, Xuzhou 221004, China; 3 School of Pharmacy, Xuzhou Medical College, Xuzhou 221004, China

**Keywords:** pyrano[3,2-*c*]quinolin-2,5(6*H*)-dione, multi-component reaction, L-proline

## Abstract

A series of 4-aryl-6-methyl-3,4-dihydro-2*H*-pyrano[3,2-*c*]quinolin-2,5(6*H*)-diones were synthesized via the three-component reactions of aromatic aldehydes, 4-hydroxy-1-methylquinolin-2(1*H*)-one, and Meldrum’s acid catalyzed by L-proline. The structures of the products were identified by spectroscopic analysis. A mechanism for this three-component reaction catalyzed by L-proline was proposed.

## 1. Introduction

Multicomponent reactions (MCRs) play an increasingly important role in organic and medical chemistry for their high degree of atom economy, convergence, productivity, easy execution, generally excellent yields and broad applications in combinatorial chemistry [1]. MCRs are highly efficient strategies to achieve the rapid assembly of complex products, especially sequential carbon-carbon and carbon-heteroatom bond-forming reactions in the area of heterocycles and natural products [2,3,4].

Pyranoquinolines and their derivatives have been reported to possess antidiabetic activities [5] and pure calcium channel blocking activities [6]. A few methods have been reported for the synthesis of pyranoquinoline derivatives from 3-oxopropanoic acid [7], or by a MCRs of malononitrile (or cyanoacetate) with aldehydes and 4-hydroxyl-1,2-dihydroquinolin-2-one (or 8-hydroxyquinoline) in the presence of KF-Al_2_O_3_ [8,9,10], TEA [11] and NEt_3_ [12] as catalysts under reflux or microwave irradiation conditions.

In recent years, the use of L-proline in different organic reactions has drawn much interest because of its experimental simplicity, ease of handing, cost effectiveness, and excellent solubility in water and organic solvents [13,14,15,16,17]. L-proline is a very efficient catalyst in transformations such as enamine-based direct catalytic asymmetric aldol condensations [18,19], Mannich reactions [20,21], Diels-Alder reactions [22] and Michael additions [23]. Proline has also been used as a catalyst for two-carbon homologation and in various one-pot multicomponent reactions [24,25,26]. As continuation of our interest in developing new methodologies for the preparation of heterocyclic compounds, herein we report a mild and highly efficient protocol for the synthesis of 3,4-dihydro-2*H*-pyrano[3,2-*c*]quinolin-2,5(6*H*)-diones catalyzed by L-proline.

## 2. Results and Discussion

**Scheme 1:**
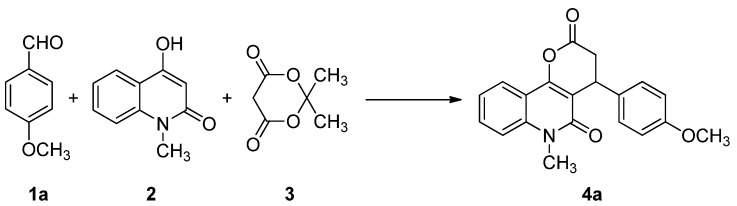
The model reaction.

Initially, the three-component reaction of 4-methoxybenzaldehyde (**1a**), 4-hydroxy-1-methylquinolin-2(1*H*)-one (**2**), and Meldrum’s acid (**3**) was investigated as a model reaction to establish the feasibility of the strategy and to optimize the reaction conditions (Scheme 1). The effects of solvents and catalyst loading were evaluated for this model reaction, and the results are summarized in Table 1.

**Table 1:** Optimization of reaction conditions.

Entry	Solvents	Catalyst (mol%)	Temperature (°C)	Time (h)	Yield (%)
1	EtOH	No	reflux	5	45
2	EtOH	L-proline (10%)	reflux	1	91
3	CH_3_CN	L-proline (10%)	reflux	2	70
4	CHCl_3_	L-proline (10%)	reflux	2	63
5	HOAc	L-proline (10%)	100	2	80
6	DMF	L-proline (10%)	100	2	52
7	H_2_O	L-proline (10%)	reflux	4	45
8	EtOH	L-proline (5%)	reflux	2	75
9	EtOH	L-proline (15%)	reflux	1	90
10	EtOH	L-proline (20%)	reflux	1	91

It was found that when the reaction was carried out without any catalyst, only a modest amount of product was obtained, even after 5 h (Table 1, entry 1). When the reaction was conducted in the presence of L-proline (10 mol%) in ethanol, the target compound **4a** was obtained in 91% yield (Table 1, entry 2). Other solvents were also used in this reaction. The results indicated that ethanol provided much better results than acetonitrile, chloroform, acetic acid, *N,N*-dimethylformamide (DMF), and water (Table 1, entries 2–7).

**Scheme 2:**
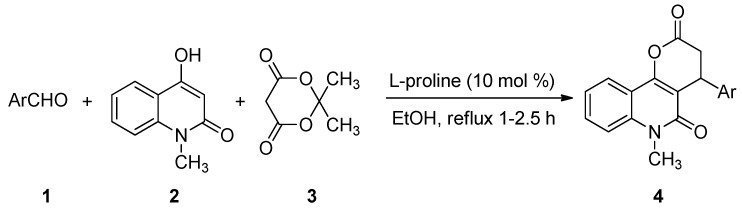
Synthesis of 4-aryl-6-methyl-3,4-dihydro-2*H*-pyrano[3,2-*c*]quinolin-2,5(6*H*)-diones **4**.

To optimize the catalyst loading, 5 mol%, 10 mol%, 15 mol%, and 20 mol% of L-proline were tested in the reactions, respectively (Table 1, entries 2, and 8–10). A 10 mol% loading of L-proline was sufficient to efficiently push the reaction forward, while 5 mol% of L-proline was not enough. Higher amounts of L-proline did not lead to significant changes in the reaction yields. With these optimum conditions in hand, a series of 4-aryl-6-methyl-3,4-dihydro-2*H*-pyrano[3,2-*c*]quinolin-2,5(6*H*)-dione derivatives were synthesized via three-component reactions of aromatic aldehydes **1**, 4-hydroxy-1-methylquinolin-2(1*H*)-one (**2**), and Meldrum’s acid (**3**) in ethanol in the presence of L-proline (Scheme 2). The results sare summarized in Table 2.

**Table 2:** Synthesis of pyrano[3,2-*c*]quinoline-2,5-diones **4** catalyzed by L-proline

Entry	Ar	Product	Time (h)	Yield (%)
1	4-CH_3_OC_6_H_4_	**4a**	2	91
2	4-BrC_6_H_4_	**4b**	1	94
3	4-HOC_6_H_4_	**4c**	1	95
4	4-(CH_3_)_2_NC_6_H_4_	**4d**	1.5	93
5	Thiophen-2-yl	**4e**	2	92
6	3-ClC_6_H_4_	**4f**	1.5	95
7	4-ClC_6_H_4_	**4g**	1	92
8	4-FC_6_H_4_	**4h**	2	90
9	3,4-(CH_3_)_2_C_6_H_3_	**4i**	2.5	91

As shown in Table 2, this protocol could be applied not only to the aromatic aldehydes with electron-withdrawing groups (such as halides), but also to the aromatic aldehydes with electron-donating groups (such as alkyl and hydroxy groups) therefore, we concluded that the electronic nature of the substituents of aromatic aldehydes has no significant effect on this reaction.

The structures of the compound **4** were identified by their spectroscopic analysis. Thus, the infrared (IR) spectra of compound **4** measured in potassium bromide pellets showed two bands for the stretching vibrations of the C=O groups at 1,653–1,658 and 1,773–1,783 cm^−1^, respectively. In the ^1^H-NMR spectra of compounds **4** measured in dimethyl sulfoxide-*d*_6_, the quinoline N-CH_3_ proton signals at 3.64–3.69 ppm, the CH_2_ proton signals at 2.89–3.11 and 3.42–3.55 ppm, the CH proton signals at 4.44–4.81 ppm, and the aromatic proton signals at 6.64–7.95 ppm were observed.

**Scheme 3:**
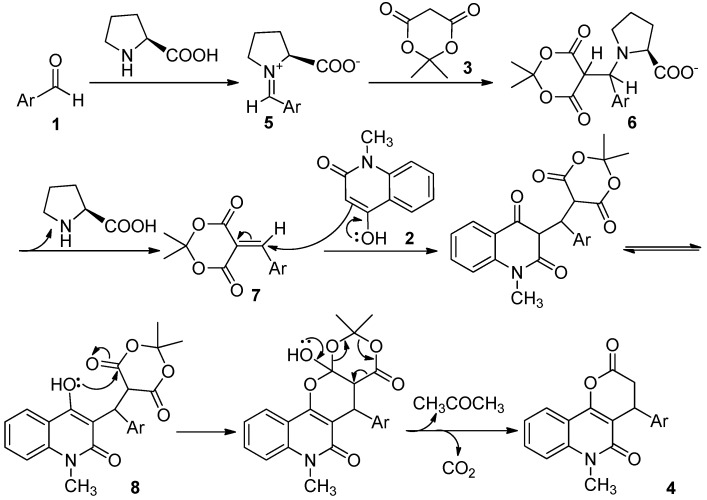
Proposed mechanism for the formation of pyrano[3,2-*c*]quinoline-2,5-diones **4**.

Although the detailed mechanism of above reaction remains to be fully clarified, the formation of compounds **4** could be explained by a reaction sequence presented in Scheme 3. We propose that the reaction procees via a reaction sequence of condensation, addition, cyclization, and elimination. We suggest that L-proline may catalyze the formation of iminium ion **5** in a reversible reaction with aldehydes **1**. The higher reactivity of the iminium ion compared with the carbonyl species could facilitate Knovenagel condensation between aldehyde **1** and Meldrum’s acid (**3**) via intermediate **6**, and after elimination of L-proline, compound **7** might be produced as an intermediate. Then, intermediate **7** is attacked via Michael addition of 4-hydroxy-1-methylquinolin-2(1*H*)-one (**2**) to give the intermediate **8**, which is followed by the cycloaddition and loss of acetone and carbon dioxide to form the desired products **4**.

## 3. Experimental

### General

All reagents were purchased from commercial sources and used without further purification. Melting points were measured on an Electrothermal XT-5 apparatus. IR spectra were recorded on a Varian F-1000 spectrometer in KBr with absorptions given in cm^−1^. ^1^H-NMR spectra were determined on a Varian Invoa-400 MHz spectrometer in DMSO-*d*_6_ solutions. *J* values are in Hz. Chemical shifts are expressed in ppm downfield from internal standard TMS. HRMS data were obtained using a TOF-MS instrument (Micromass Inc., Manchester, UK).

General Procedure for the Synthesis of 4-Aryl-6-methyl-3,4-dihydro-2H-pyrano[3,2-c]quinolin-2,5(6*H*)-diones **4**

A mixture of aromatic aldehyde **1** (1 mmol), 4-hydroxy-1-methylquinolin-2(1*H*)-one (**2**, 0.175 g, 1 mmol), Meldrum’s acid (**3**, 1 mmol), L-proline (0.0115 g, 0.1 mmol) and ethanol (2 mL) in a 50 mL round bottom flask was stirred at 80 °C for 1–2.5 h. At the end of the reaction, the mixture was cooled to room temperature. The precipitate was collected by filtration and purified by recrystallization from EtOH and DMF to give products **4**.

*4-(4-Methoxyphenyl)-6-methyl-3,4-dihydro-2H-pyrano[3,2-c]quinoline-2,5(6H)-dione* (**4a**). White solid; m.p. 192–194 °C. IR: 3041 (C-H Ar), 2924 (C-H aliph), 1782 (C=O), 1654 (C=O), 1600 (C=C), 1514 (ArC=C), 1380 (C-H CH_3_), 1252 (C-O); ^1^H-NMR: δ (ppm) 2.92 (d, *J* = 16.0 Hz, 1H, CH_2_), 3.46 (dd, *J*_1_ = 7.6 Hz, *J*_2_ = 16.0 Hz, 1H, CH_2_), 3.64 (s, 3H, CH_3_), 3.69 (s, 3H, OCH_3_), 4.51 (d, *J* = 7.2 Hz, 1H, CH), 6.86 (d, *J* = 8.4 Hz, 2H, ArH), 7.09 (d, *J* = 8.8 Hz, 2H, ArH), 7.40 (t, *J*_1_ = 8.0 Hz, *J*_2_ = 7.2 Hz, 1H, ArH), 7.64 (d, *J* = 8.8 Hz, 1H, ArH), 7.75 (t, *J*_1_ = 7.6 Hz, *J*_2_ = 7.2 Hz, 1H, ArH), 7.93 (d, *J* = 8.0 Hz, 1H, ArH); HRMS calculated for C_20_H_17_NO_4_ [M]^+^: 335.1158, Found 335.1158.

*4-(4-Bromophenyl)-6-methyl-3,4-dihydro-2H-pyrano[3,2-c]quinoline-2,5(6H)-dione* (**4b**). White solid; m.p. 239–241 °C. IR: 3024 (C-H Ar), 2928 (C-H aliph), 1778 (C=O), 1655 (C=O), 1596 (C=C), 1503 (ArC=C), 1384 (C-H CH_3_), 1209 (C-O); ^1^H-NMR: δ (ppm) 2.96 (d, *J* = 16.0 Hz, 1H, CH_2_), 3.52 (dd, *J*_1_ = 7.6 Hz, *J*_2_ = 16.0 Hz, 1H, CH_2_), 3.65 (s, 3H, CH_3_), 4.56 (d, *J* = 7.2 Hz, 1H, CH), 7.16 (d, *J* = 8.4 Hz, 2H, ArH), 7.41 (t, *J*_1_ = 7.6 Hz, *J*_2_ = 7.2 Hz, 1H, ArH), 7.51 (d, *J* = 8.4 Hz, 2H, ArH), 7.66 (d, *J* = 8.8 Hz, 1H, ArH), 7.76 (t, *J*_1_ = 7.6 Hz, *J*_2_ = 8.4 Hz, 1H, ArH), 7.95 (d, *J* = 8.0 Hz, 1H, ArH); HRMS calculated for C_19_H_14_NO_3_^79^Br [M]^+^: 383.0157, Found 383.0148.

*4-(4-Hydroxyphenyl)-6-methyl-3,4-dihydro-2H-pyrano[3,2-c]quinoline-2,5(6H)-dione* (**4c**). White solid; m.p. > 300 °C. IR: 3282 (O-H), 3013 (C-H Ar), 1781 (C=O), 1653 (C=O), 1600 (C=C), 1517 (ArC=C), 1385 (C-H CH_3_), 1202 (C-O); ^1^H-NMR: δ (ppm) 2.90 (d, *J* = 16.0 Hz, 1H, CH_2_), 3.43 (dd, *J*_1_ = 7.2 Hz, *J*_2_ = 15.6 Hz, 1H, CH_2_), 3.65 (s, 3H, CH_3_), 4.56 (d, *J* = 6.8 Hz, 1H, CH), 6.69 (d, *J* = 8.4 Hz, 2H, ArH), 6.97 (d, *J* = 8.4 Hz, 2H, ArH), 7.40 (t, *J*_1_ = 7.6 Hz, *J*_2_ = 7.6 Hz, 1H, ArH), 7.64 (d, *J* = 8.8 Hz, 1H, ArH), 7.75 (t, *J*_1_ = 8.4 Hz, *J*_2_ = 7.6 Hz, 1H, ArH), 7.93 (d, *J* = 8.0 Hz, 1H, ArH), 9.37 (s, 1H, OH); HRMS calculated for C_19_H_15_NO_4_ [M]^+^: 321.1001, Found 321.1001.

*4-(4-Dimethylaminophenyl)-6-methyl-3,4-dihydro-2H-pyrano[3,2-c]quinoline-2,5(6H)-dione* (**4d**). White solid; m.p. 220–222 °C. IR: 2981 (C-H aliph), 2798 (C-H aliph), 1773 (C=O), 1654 (C=O), 1598 (C=C), 1519 (ArC=C), 1382 (C-H CH_3_), 1208 (C-O); ^1^H-NMR: δ (ppm) 2.83 (s, 6H, 2 × CH_3_), 2.90 (d, *J* = 16.0 Hz, 1H, CH_2_), 3.42 (dd, *J*_1_ = 7.6 Hz, *J*_2_ = 16.0 Hz, 1H, CH_2_), 3.65 (s, 3H, CH_3_), 4.44 (d, *J* = 6.8 Hz, 1H, CH), 6.64 (d, *J* = 8.0 Hz, 2H, ArH), 6.98 (d, *J* = 8.4 Hz, 2H, ArH), 7.40 (t, *J*_1_ = 8.0 Hz, *J*_2_ = 7.2 Hz, 1H, ArH), 7.64 (d, *J* = 8.8 Hz, 1H, ArH), 7.73 (t, *J*_1_ = 7.6 Hz, *J*_2_ = 8.0 Hz, 1H, ArH), 7.93 (d, *J* = 8.0 Hz, 1H, ArH); HRMS calculated for C_21_H_20_N_2_O_3_ [M]^+^: 348.1474, Found 348.1478.

*6-Methyl-4-(thiophen-2-yl)-3,4-dihydro-2H-pyrano[3,2-c]quinoline-2,5(6H)-dione* (**4e**). White solid; m.p. 243–244 °C. IR: 3040 (C-H Ar), 2947 (C-H aliph), 1774 (C=O), 1658 (C=O), 1596 (C=C), 1587 (ArC=C), 1213 (C-O); ^1^H-NMR: δ (ppm) 3.11 (d, *J* = 15.2 Hz, 1H, CH_2_), 3.51–3.55 (m, 1H, CH_2_), 3.69 (s, 3H, CH_3_), 4.81 (d, *J* = 1.2 Hz, 1H, CH), 6.93 (d, *J* = 18.0 Hz, 2H, ArH), 7.39 (d, *J* = 5.6 Hz, 2H, ArH), 7.67 (s, 1H, ArH), 7.76 (s, 1H, ArH), 7.92 (s, 1H, ArH); HRMS calculated for C_17_H_13_NO_3_S [M]^+^: 311.0616, Found 311.0612.

*4-(3-Chlorophenyl)-6-methyl-3,4-dihydro-2H-pyrano[3,2-c]quinoline-2,5(6H)-dione *(**4f**). White solid; m.p. 232–233 °C. IR: 3054 (C-H Ar), 2939 (C-H aliph), 1783 (C=O), 1654 (C=O), 1594 (C=C), 1387 (C-H CH_3_), 1205 (C-O); ^1^H-NMR: δ (ppm) 2.99 (d, *J* = 16.0 Hz, 1H, CH_2_), 3.52 (dd, *J*_1_ = 7.6 Hz, *J*_2_ = 16.4 Hz, 1H, CH_2_), 3.65 (s, 3H, CH_3_), 4.59 (d, *J* = 6.8 Hz, 1H, CH), 7.11 (d, *J* = 5.6 Hz, 1H, ArH), 7.30–7.36 (m, 3H, ArH), 7.41 (t, *J*_1_ = 7.6 Hz, *J*_2_ = 7.6 Hz, 1H, ArH), 7.65 (d, *J* = 8.4 Hz, 1H, ArH), 7.76 (t, *J*_1_ = 7.2 Hz, *J*_2_ = 7.6 Hz, 1H, ArH), 7.94 (d, *J* = 8.0 Hz, 1H, ArH); HRMS calculated for C_19_H_14_NO_3_^35^Cl [M]^+^: 339.0662, found 339.0648.

*4-(4-Chlorophenyl)-6-methyl-3,4-dihydro-2H-pyrano[3,2-c]quinoline-2,5(6H)-dione *(**4g**). White solid; m.p. 227–228 °C. IR: 3083 (C-H Ar), 2935 (C-H aliph), 1779 (C=O), 1657 (C=O), 1593 (C=C), 1385 (C-H CH_3_), 1207 (C-O); ^1^H-NMR: δ (ppm) 2.97 (d, *J* = 16.0 Hz, 1H, CH_2_), 3.52 (dd, *J*_1_ = 7.6 Hz, *J*_2_ = 16.0 Hz, 1H, CH_2_), 3.65 (s, 3H, CH_3_), 4.58 (d, *J* = 7.2 Hz, 1H, CH), 7.22 (d, *J* = 8.4 Hz, 2H, ArH), 7.37 (d, *J* = 8.4 Hz, 2H, ArH), 7.42 (d, *J* = 7.6 Hz, 1H, ArH), 7.66 (d, *J* = 8.8 Hz, 1H, ArH), 7.76 (t, *J*_1_ = 7.2 Hz, *J*_2_ = 8.4 Hz, 1H, ArH), 7.95 (d, *J* = 8.0 Hz, 1H, ArH); HRMS calculated for C_19_H_14_NO_3_^35^Cl [M]^+^: 339.0662, Found 339.0654.

*4-(4-Fluorophenyl)-6-methyl-3,4-dihydro-2H-pyrano[3,2-c]quinoline-2,5(6H)-dione *(**4h**). White solid; m.p. 230–231 °C. IR: 3076 (C-H Ar), 2923 (C-H aliph), 1777 (C=O), 1656 (C=O), 1598 (C=C), 1506 (ArC=C), 1386 (C-H CH_3_), 1214 (C-O); ^1^H-NMR: δ (ppm) 2.96 (d, *J* = 16.0 Hz, 1H, CH_2_), 3.50 (dd, *J*_1_ = 7.6 Hz, *J*_2_ = 16.0 Hz, 1H, CH_2_), 3.64 (s, 3H, CH_3_), 4.58 (d, *J* = 7.2 Hz, 1H, CH), 7.13 (t, *J*_1_ = 8.4 Hz, *J*_2_ = 8.8 Hz, 2H, ArH), 7.23 (t, *J*_1_ = 7.6 Hz, *J*_2_ = 8.4 Hz, 2H, ArH), 7.40 (t, *J*_1_ = 7.6 Hz, *J*_2_ = 7.6 Hz, 1H, ArH), 7.64 (d, *J* = 8.4 Hz, 1H, ArH), 7.75 (t, *J*_1_ = 7.2 Hz, *J*_2_ = 8.4 Hz, 1H, ArH), 7.93 (d, *J* = 8.0 Hz, 1H, ArH); HRMS calculated for C_19_H_14_NO_3_F [M]^+^: 323.0958, Found 323.0961.

*4-(3,4-Dimethylphenyl)-6-methyl-3,4-dihydro-2H-pyrano[3,2-c]quinoline-2,5(6H)-dione* (**4i**). White solid; m.p. 170–171 °C. IR: 3010 (C-H Ar), 2939 (C-H aliph), 1777 (C=O), 1654 (C=O), 1596 (C=C), 1462 (ArC=C), 1382 (C-H CH_3_), 1215 (C-O); ^1^H-NMR: δ (ppm) 2.15 (s, 6H, 2 × CH_3_ ), 2.89 (d, *J* = 16.0 Hz, 1H, CH_2_), 3.46 (dd, *J*_1_ = 8.0 Hz, *J*_2_ = 15.6 Hz, 1H, CH_2_), 3.64 (s, 3H, CH_3_), 4.48 (d, *J* = 6.4 Hz, 1H, CH), 6.84 (d, *J* = 7.6 Hz, 1H, ArH), 6.96 (s, 1H, ArH), 7.04 (d, *J* = 7.6 Hz, 1H, ArH), 7.41 (t, *J*_1_ = 8.0 Hz, *J*_2_ = 7.2 Hz, 1H, ArH), 7.64 (d, *J* = 8.4 Hz, 1H, ArH), 7.75 (t, *J*_1_ = 7.2 Hz, *J*_2_ = 8.4 Hz, 1H, ArH), 7.94 (d, *J* = 8.0 Hz, 1H, ArH); HRMS calculated for C_21_H_19_NO_3_ [M]^+^: 333.1365, Found 333.1367.

## 4. Conclusions

In summary, we have developed an efficient synthesis of 4-aryl-6-methyl-3,4-dihydro-2*H*-pyrano [3,2-*c*]quinolin-2,5(6*H*)-diones via the three-component reactions of aromatic aldehydes, 4-hydroxy-1-methylquinolin-2(1*H*)-one, and Meldrum’s acid catalyzed by L-proline. This protocol has the advantages of easy work up, mild reaction conditions, and high yields. In view of the potential biological activities of these molecules, further biomedical screening work is in progress in our laboratories.
